# Aptamer‐mediated synthesis of multifunctional nano‐hydroxyapatite for active tumour bioimaging and treatment

**DOI:** 10.1111/cpr.13105

**Published:** 2021-08-12

**Authors:** Wenqing Zhang, Ronghui Zhou, Yuting Yang, Shuanglin Peng, Dexuan Xiao, Tingting Kong, Xiaoxiao Cai, Bofeng Zhu

**Affiliations:** ^1^ Key laboratory of Shaanxi Province for Craniofacial Precision Medicine Research College of Stomatology Xi’an Jiaotong University Xi’an China; ^2^ Clinical Research Center of Shaanxi Province for Dental and Maxillofacial Diseases College of Stomatology Xi’an Jiaotong University Xi’an China; ^3^ State Key Laboratory of Oral Diseases West China Hospital of Stomatology Sichuan University Chengdu China; ^4^ Department of Oral and Maxillofacial Surgery Hospital of Stomatology Southwest Medical University Luzhou China; ^5^ Department of Stomatology Central Hospital Affiliated to Shandong First Medical University Jinan China; ^6^ Department of Forensic Genetics Multi‐Omics Innovative Research Center of Forensic Identification School of Forensic Medicine Southern Medical University Guangzhou China

**Keywords:** AS1411, biomimetic synthesis, drug carriers, dual‐model bioimaging, nano‐hydroxyapatite

## Abstract

**Objectives:**

The nano‐hydroxyapatite (nHAp) is widely used to develop imaging probes and drug carriers due to its excellent bioactivity and biocompatibility. However, traditional methods usually need cumbersome and stringent conditions such as high temperature and post‐modification to prepare the functionalized nHAp, which do not benefit the particles to enter cells due to the increased particle size. Herein, a biomimetic synthesis strategy was explored to achieve the AS1411‐targeted tumour dual‐model bioimaging using DNA aptamer AS1411 as a template. Then, the imaging properties and the biocompatibility of the synthesized AS‐nFAp:Gd/Tb were further investigated.

**Materials and methods:**

The AS‐nFAp:Gd/Tb was prepared under mild conditions through a one‐pot procedure with AS1411 as a template. Besides, the anticancer drug DOX was loaded to AS‐nFAp:Gd/Tb so as to achieve the establishment of a multifunctional nano‐probe that integrated the tumour diagnosis and treatment. The AS‐nFAp:Gd/Tb was characterized by transmission electron microscopy (TEM), energy disperse X‐ray Spectroscopy (EDS) mapping, X‐ray photoelectron spectroscopy (XPS) spectrum, X‐ray diffraction (XRD), fourier‐transformed infrared (FTIR) spectroscopy, capillary electrophoresis analyses, zeta potential and particle sizes. The in vitro magnetic resonance imaging (MRI) and fluorescence imaging were performed on an MRI system and a confocal laser scanning microscope, respectively. The potential of the prepared multifunctional nHAp for a targeted tumour therapy was investigated by a CCK‐8 kit. And the animal experiments were conducted on the basis of the guidelines approved by the Animal Care and Use Committee of Sichuan University, China.

**Results:**

In the presence of AS1411, the as‐prepared AS‐nFAp:Gd/Tb presented a needle‐like morphology with good monodispersity and improved imaging performance. Furthermore, due to the specific binding between AS1411 and nucleolin up‐expressed in cancer cells, the AS‐nFAp:Gd/Tb possessed excellent AS1411‐targeted fluorescence and MRI imaging properties. Moreover, after loading chemotherapy drug DOX, in vitro and in vivo studies showed that DOX@AS‐nFAp:Gd/Tb could effectively deliver DOX to tumour tissues and exert a highly effective tumour inhibition without systemic toxicity compared with pure DOX.

**Conclusions:**

The results indicated that the prepared multifunctional nHAp synthesized by a novel biomimetic strategy had outstanding capabilities of recognition and treatment for the tumour and had good biocompatibility; hence, it might have a potential clinical application in the future.

## 1 INTRODUCTION

The preparation of biodegradable and multifunctional nanoparticles with excellent biocompatibility, superior imaging and therapeutic capabilities is of great significance for improving the diagnosis and treatment of tumours.^1^, ^2^, ^3^ In recent years, various nanotechnologies have been designed in the field of biomedicine and have received extensive attention.^4^, ^5^, ^6^, ^7^ The hydroxyapatite (HAp, Ca_10_(PO_4_)_6_(OH)_2_) is a primary inorganic composition of mammalian hard tissues (bone and teeth), as a biomaterial scaffold with good biocompatibility and biodegradability, the nano‐HAp (nHAp) and F‐substituted nHAp (nFAp) were widely used in the biomedical field.^8^, ^9^, ^10^, ^11^ Recently, a variety of bioimaging systems based on nHAp have been prepared for dual/multi‐mode tracking.^12^, ^13^, ^14^ In general, the construction of nHAp imaging probes is achieved by post‐modifying various imaging contrast agents through physical or chemical interaction such as gold nanoparticles, quantum dots and carbon dots. However, the tedious post‐modification is not conductive to the good monodispersity of nanoparticles.^12^, ^15^ With the feature of unique hexagonal structure and space group, HAp has the ability to easily replace Ca^2+^ in the crystal lattice by lanthanide ions (Ln^3+^) (eg Eu^3+^, Gd^3+^, Tb^3+^), thereby endowing HAp with specific imaging performance.^16^, ^17^, ^18^ Therefore, the dual/multi‐modal bioimaging is easily achieved by co‐doping two or more Ln^3+^ ions, and the tedious modification process is reduced simultaneously. Stringent conditions such as high temperature and specific pH values are usually required for the synthesis of doped‐nHAp,^19^, ^20^ which are not beneficial to the internalization of the particles in the cells due to the increased particle size.^16^ Hence, there is an urgent need for developing a simple method to synthesize multifunctional nHAp under milder conditions for clinical applications.

Recently, biological macromolecules, such as polysaccharides and natural rubber latex, have been widely used as templates in the biomimetic methods to synthesize nHAp nanoparticles (NPs) under mild conditions, because the nHAp NPs synthesized by these methods have better physical, chemical and biological properties than traditional methods.^21^, ^22^ In addition, nHAp can be directly functionalized in the biomimetic methods to avoid the post‐modification of nHAp .^23^ For example, based on the affinity of hyaluronan (HA) to CD44 overexpressed in tumour cells, a HA‐mediated Eu/Ba co‐doped nFAp was constructed, and in comparison with pure nFAp:Eu/Ba synthesized by traditional methods, the prepared HA@nFAp:Eu/Ba possessed the ability to target tumours and improved bioimaging performance.^24^ Moreover, a synthetic strategy was developed to synthesize nHAp (tHA) with polydopamine (pDA) as a template, and nHAp was then introduced into polycaprolactone (PCL) to prepare tHA/PCL, demonstrating an enhanced osteogenic ability and biocompatibility compared with traditional nHAp equipped with PCL or pure PCL.^25^ Consequently, the biomimetic synthesis strategy is feasible to prepare a functionalized nHAp on one‐pot procedure under mild conditions.

The DNA aptamer AS1411 is widely employed as a tumour‐targeting agent by binding with nucleolin.^26^, ^27^, ^28^, ^29^ Nucleolin is considered to be a tumour biomarker overexpressed on the surface of cancer cells (eg breast cancer and melanoma),^30^, ^31^, ^32^ which is believed to be dominant in internalization or transport of nanoparticles from the cell surface to the nucleus.^33^ Hence, we explored an AS1411‐templated strategy to synthesize co‐doped nFAp (Gd&Tb) for dual‐modal imaging targeted cancers via a one‐pot procedure in the present study. Besides, the anticancer drug DOX was loaded to nFAp so as to achieve the establishment of a multifunctional nano‐probe that integrated the tumour diagnosis and treatment. A series of characterization techniques were performed to prove the successful synthesis of this functionalized nFAp, and the synthetic conditions were further optimized. The AS‐nFAp:Gd/Tb synthesized under the AS1411‐templated method has better abilities of imaging and recognizing the tumour compared with AS1411‐free nHAp:Gd/Tb. In vitro and in vivo experiments demonstrated that the synthesized AS‐nFAp:Gd/Tb could be used as a drug carrier to target tumour imaging and treatment. Meanwhile, the reduced systemic toxicity of the chemotherapy drug indicated its greater potential in clinical applications.

## 2 MATERIALS AND METHODS

### 2.1 Materials

Raw materials including calcium nitrate tetrahydrate (Ca(NO_3_)_2_•4H_2_O), sodium phosphate dodecahydrate (Na_3_PO_4_.12H_2_O) and sodium fluoride (NaF) were purchased from Chengdu Kelong Chemical Co., Ltd., China. The gadolinium nitrate hexahydrate (Gd(NO_3_)_3_•6H_2_O) and terbium nitrate pentahydrate (Tb(NO_3_)_3_•5H_2_O) were bought from Aladdin Chemical Inc, China. The AS1411 aptamer (GGTGGTGGTGGTTGTGGTGGTGGTGG) was synthesized by Sangon Biotech, Co., Ltd., China. The doxorubicin hydrochloride (C_27_H_29_NO_11_·HCl, DOX•HCl) was bought from Beijing Solarbio Science & Technology Co., Ltd., China. The Dulbecco's modified Eagle's medium (DMEM), RPMI 1640 medium, foetal bovine serum (FBS) and 0.25% trypsin‐EDTA were obtained from GIBCO, Invitrogen Co., Carlsbad, USA. A Cell Counting Kit 8 (CCK‐8) was obtained from Dojindo Laboratories, Japan, and 4′,6‐Diamidino‐2‐phenylindole (DAPI) and phalloidin were purchased from Shanghai Beyotime Biotechnology Co., Ltd., China. The ultrapure water (18.2 MΩ cm^−1^) obtained from a purification system (Direct 8, Milli‐Q) was utilized though the whole experiments. All chemical reagents in this study were of analytical grade.

**SCHEME 1:**
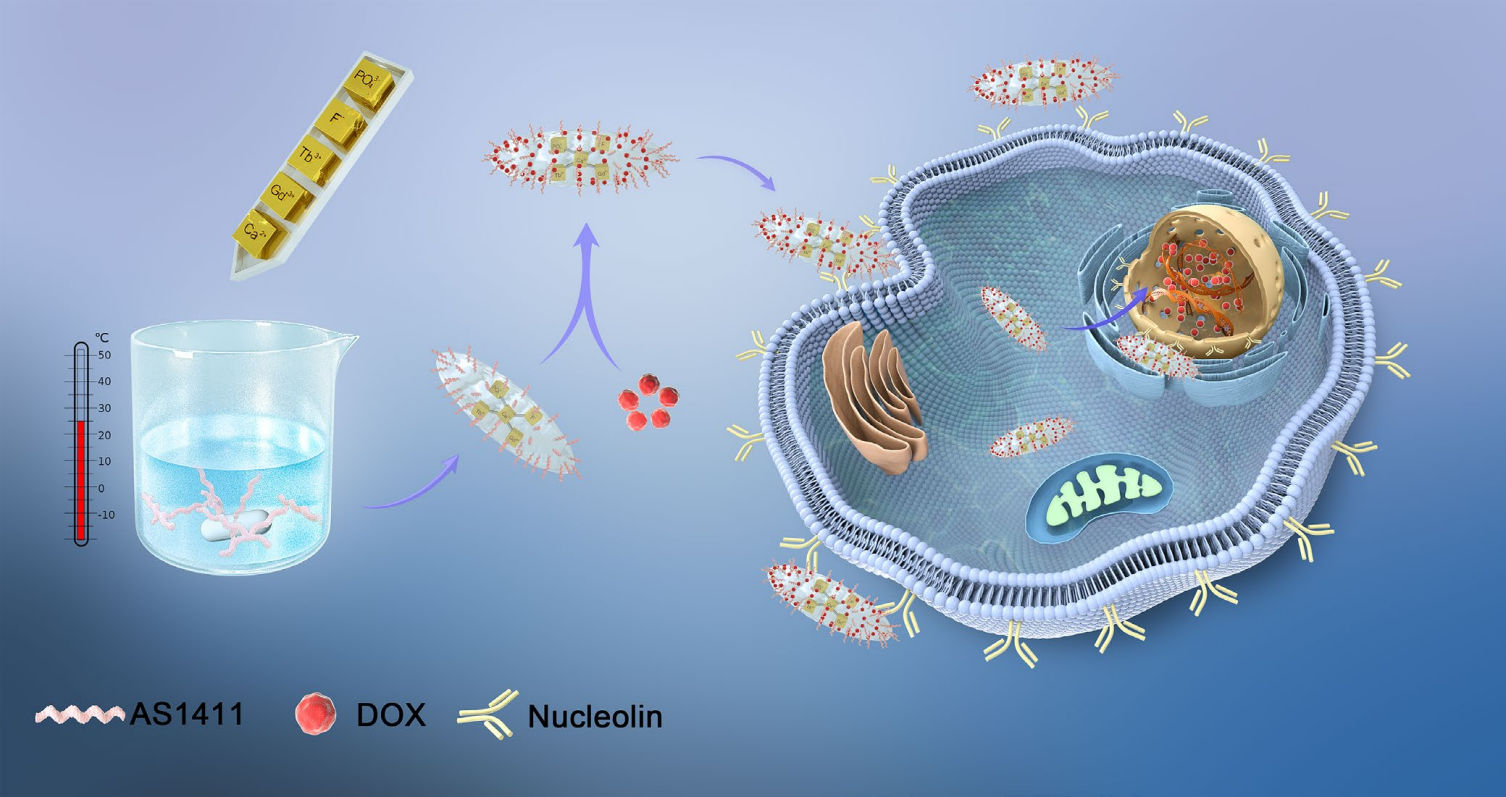
Schematic illustration of AS‐nFAp:Gd/Tb preparation and application

### 2.2 Characterization

A transmission electron microscopy (TEM) was used to observe the morphology of samples (JEM‐2100F, JEOL). The elemental composition was analysed via both energy disperse X‐ray Spectroscopy (EDS) mapping on TEM and X‐ray photoelectron spectroscopy (XPS) spectrum (AXIS Ultra DLD). The X‐ray diffraction (XRD) obtained from a PANalytical Empyrean instrument with Cu Kα (λ = 1.5406 Å) was used to detect the phase composition. The fourier‐transformed infrared (FTIR) spectroscopy (Nicolet 6700) was carried out to identify the functional groups of samples in the examination range of 4000‐400 cm^‐1^. The capillary electrophoresis analyses were conducted on a Qsep100 Bio‐Fragment Analyzer (BiOptic). The zeta potential and particle size were obtained from a Zetasizer Nano ZS90. A fluorescence spectrometer (Horiba, FL‐4) was utilized to analyse the fluorescence spectra. The in vitro MRI and fluorescence imaging were performed on an MRI system (HT‐MRSI60‐50A, HTNMR) and a confocal laser scanning microscope (CLSM, AIR MP+), respectively.

### 2.3 Synthesis of AS‐nFAp:Gd/Tb

The co‐doped nFAp (the concentration molar ratio of Ca/AS1411 was 10 000, 5000, 2000, 1000 and 500, respectively) was synthesized through a co‐precipitation method with AS1411 as a template. Firstly, AS1411 (100 μmol/L) was dissolved in ultrapure water. Then, 50 μL Ca(NO_3_)_2_ solution (1 mol/L) was added to the systems of AS1411 with different content under string. Whereafter, Gd(NO_3_)_3_ solution (1 mol/L) and Tb(NO_3_)_3_ solution (1 mol/L) (the concentration molar ratio of Gd&Tb/Ca = 5%, 10%, 15% and 20%) were successively added to the reaction system. After stirring for 10 minutes, NaOH solution (1 mol/L) was used to regulate the pH value of the reaction solution to 10, and then 9 μL of NaF (1 mol/L) and 50 μL of Na_3_PO_4_ (0.6 mol/L) solution were put dropwise into the reaction system. The total volume was fixed to 1 mL, and the whole reaction system was required to be stirred at room temperature for different times (2, 6, 12 and 24 hours). Finally, the precipitate was collected after washing with ultrapure water for three times by centrifugation (8000 rpm, 5 minutes) and dispersed in the aqueous solution at the end of the reaction. For comparison, the AS1411‐Free nFAp:Gd/Tb was synthesized by the same procedure described above except for the use of AS1411.

### 2.4 In vitro MRI imaging and photostability

The AS‐nFAp:Gd/Tb was dispersed in the aqueous solution with different concentrations of Gd^3+^ (0.2‐2 mmol/L). Then, the MRI images of the above samples were acquired by an MRI system. The fluorescence intensity of aqueous solution of AS‐nFAp:Gd/Tb was measured at different time points within 1 hour by a fluorescence spectrophotometer to test the photostability of AS‐nFAp:Gd/Tb, with the fluorescent dye rhodamine as a control.

### 2.5 In vitro fluorescence imaging of cells

The SCC‐25 (human tongue squamous cell carcinoma cell line) and L929 (mouse fibroblast cell line) cells were cultured in confocal dishes with 1 × 10^5^ cells/well at 37°C under 5% CO_2_ for 24 hours. The culture mediums of above cells were DMEM and RPMI 1640 medium containing 10% FBS and 1% penicillin‐streptomycin solution, respectively. After removing the culture medium, SCC‐25 and L929 cells were incubated with 100 μg/mL AS‐nFAp:Gd/Tb for 2 time periods (6 hours and 12 hours), respectively. Then, the cells were fixed with 4% paraformaldehyde, and the cytoskeletons were stained with phalloidin after washing with phosphate buffer solution (PBS).^34^ Finally, fluorescence imaging was obtained by a CLSM. Meanwhile, the cells were also incubated with AS1411‐free nFAp:Gd/Tb (100 μg/mL) for 12 hours to observe the fluorescence imaging as a comparison.

### 2.6 Preparation of DOX@AS‐nFAp:Gd/Tb

The AS‐nFAp:Gd/Tb was used as a drug carrier to load DOX. In general, an equal volume of DOX (100 μg/mL) was added into the AS‐nFAp:Gd/Tb aqueous solution (5 mg/mL), and then stirred for 6 hours under room temperature. Then, the complex (DOX@AS‐nFAp:Gd/Tb) was acquired after washing with ultrapure water for three times by centrifugation (8000 rpm, 5 minutes) to remove free chemicals.

### 2.7 In vitro drug loading and release

The drug loading efficiency and loading content were calculated by a UV‐vis spectroscopy. The loading content of DOX in DOX@AS‐nFAp:Gd/Tb was obtained from a standard dilution curve of DOX based on the absorbance measured under 485 nm at different concentrations. In order to explore the drug release behaviour of DOX@AS‐nFAp:Gd/Tb, the same amount of which was placed in three dialysis bags and incubated in PBS with diverse pH environments (pH = 5.5, 6.5 and 7.4). The release system was incubated at 37°C with slightly stirring. At several time intervals, equal amounts of liquid were withdrawn from the three release systems and substituted with the corresponding PBS. The amount of DOX released from DOX@AS‐nFAp:Gd/Tb was measured on a UV‐vis spectroscopy.

### 2.8 Cell viability and cell apoptosis analyses

A CCK‐8 assay was conducted to test the cell viability of different cell lines, as indicated in the protocol.^35^ Firstly, SCC‐25 and L929 cells seeded in 96‐well plates were cultured overnight. Then, the cells were treated with mediums containing AS‐nFAp:Gd/Tb, DOX and DOX@AS‐nFAp:Gd/Tb at gradient concentrations for 24 hours (the content of DOX in DOX@AS‐nFAp:Gd/Tb group was equivalent to pure DOX group). Afterwards, the fresh medium containing 10% CCK‐8 solution was co‐cultured with cells under the atmosphere of 5% CO_2_ at 37°C after washing with PBS for 3 times. About 1 hour later, the optical density (OD) values of 96‐well plates were detected at 450 nm by a microplate reader (Varioskan Flash, Thermo Fisher Scientific). In order to conduct the cell apoptosis analysis, the SCC‐25 and L929 cultured in confocal dishes overnight were incubated with AS‐nFAp:Gd/Tb, pure DOX and DOX@AS‐nFAp:Gd/Tb at the same concentration for 24 hours (AS‐nFAp:Gd/Tb: 250 μg/mL, DOX: 5 μg/mL, DOX@AS‐nFAp:Gd/Tb: 250 μg/mL, the content of DOX in DOX@AS‐nFAp:Gd/Tb group was equivalent to pure DOX group). Cells without any treatment were regarded as a control. Then, cells were fixed with 4% paraformaldehyde and treated by 0.05% TritonX‐100 to penetrate the cell membrane. Cells were further blocked with goat serum, and PBS was required to wash cells at each operation interval. The primary antibodies against Bcl‐2, Bax and Caspase‐3 were used to cultivate cells overnight at 4°C, and the second antibody was applied the next day. The cell nucleus was stained with DAPI, and the prepared samples were observed on a CLSM to monitor the relative expression of apoptosis‐related proteins.

### 2.9 In vivo anti‐tumour study

The animal experiments were conducted on the basis of the guidelines approved by the Animal Care and Use Committee of Sichuan University, China (WCHSIRB‐D‐2020‐195). The specific pathogen‐free female nude mice at 4‐5 weeks old were purchased from GemPharmatech Co., Ltd., China, and kept in a SPF‐level environment. The oral squamous cell carcinoma model was constructed by subcutaneously injecting the SCC‐25 cells (1 × 10^6^ cells/100 μL) into the right anterior armpit of each nude mouse. The tumour volume was determined as follows: volume =0.5 × length × width^2^. The mice were randomly assigned into four groups (n ≥ 3, per group) when the volume of tumour was over 50 mm^3^. Then, 100 μL of saline, AS‐nFAp:Gd/Tb (50 mg/kg), DOX (4 mg/kg) and DOX@AS‐nFAp:Gd/Tb (50 mg/kg) were injected into the paracancerous tissues of these 4 groups, respectively. The group injected with saline was set as a control group. The body weight and tumour volume of each mouse were measured and recorded every two days throughout the whole treatment period. At the end of the treatment, the mice were sacrificed, and the tumour tissues and vital organs (heart, liver, spleen, lung and kidney) were dissected and stored in 4% paraformaldehyde for TUNEL staining and H&E staining.

### 2.10 Statistical analysis

All experiments were performed for three times, and multiple group comparisons of data were carried out by one‐way analysis of variance (ANOVA) in the software GraphPad Prism 8. It was considered statistically significant when the *P* value was smaller than .05.

## 3 RESULTS AND DISCUSSION

### 3.1 Characterization of AS‐nFAp:Gd/Tb

**FIGURE 1:**
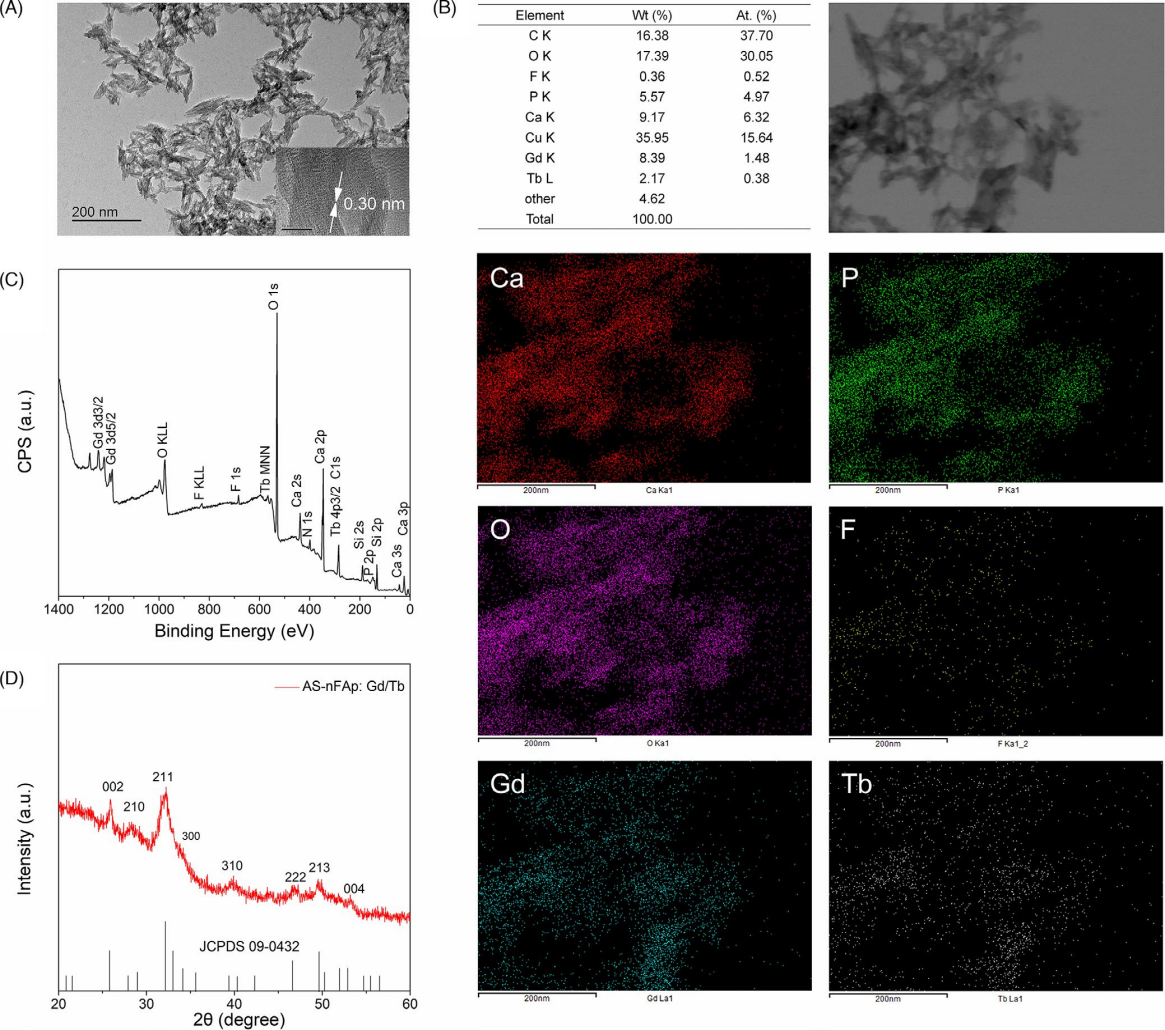
Morphology and composition of AS‐nFAp:Gd/Tb. (A), TEM image shows that the synthesized AS‐nFAp: Gd/Tb has a uniform needle‐like morphology; (B), EDS elemental mapping and patterns of AS‐nFAp: Gd/Tb; (C), XPS spectrum of AS‐nFAp:Gd/Tb; (D), XRD spectrum of AS‐nFAp:Gd/Tb and the vertical lines represent the standard diffraction peaks of nHAp (JCPDS 09‐0432)

The simplified co‐precipitation method to synthesize the AS‐nFAp:Gd/Tb probe was displayed in Scheme 1. Firstly, the negatively charged AS1411 was combined with Ca^2+^ ions through the electrostatic interaction. Subsequently, the Gd^3+^ ions and Tb^3+^ ions were doped into vacancies by replacing Ca^2+^ ions. The nHAp crystals were gradually formed through the reaction of PO_4_^3‐^ ions and Ca^2+^ ions with the addition of PO_4_^3‐^ ions in the reaction solution. In addition, fluoride was also added to nHAp to improve the uniformity of nHAp particles.^24^ As shown in the TEM image (Figure 1A), the synthesized AS‐nFAp:Gd/Tb presented a monodispersed and uniform needle‐like shape with an average size of 116 nm in length and 10 nm in diameter. The interplanar crystal spacing was about 0.30 nm. As shown in the EDS mapping (Figure 1B), the AS‐nFAp:Gd/Tb contained the elements of Ca, P, O, Gd, Tb and F, and the doped elements (Gd&Tb) were uniformly distributed in the nanoparticles. Moreover, as confirmed in the XPS spectrum (Figure 1C), the Gd^3+^ ions and Tb^3+^ ions were successfully co‐doped into the prepared AS‐nFAp: Gd/Tb. The diffraction peaks of AS‐nFAp:Gd/Tb in the XRD spectra (Figure 1D) were the same with the standard diffraction peaks of nHAp (JCPDS 09‐0432) at planes of (002), (210), (211), (300), (310), (222), (213) and (004).^36^

**FIGURE 2:**
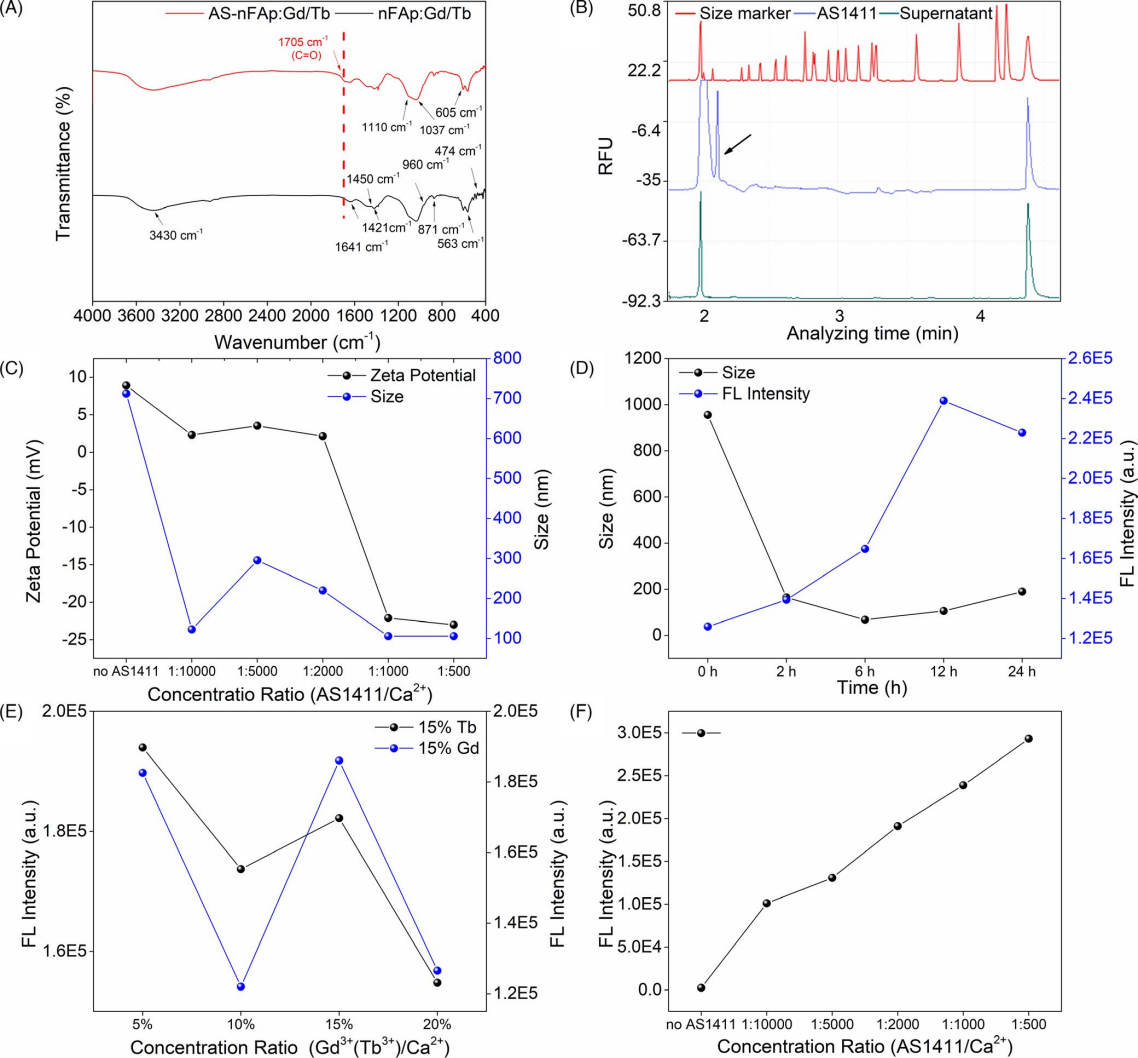
Characterization of AS‐nFAp:Gd/Tb. (A), FTIR spectra of AS‐nFAp:Gd/Tb and AS1411‐free nFAp:Gd/Tb NPs; (B), Electropherograms of AS‐nFAp:Gd/Tb NPs; (C), Zeta potential values and particle sizes of AS‐nFAp:Gd/Tb with different concentration molar ratio of AS1411 to Ca^2+^; (D), The particle sizes and the fluorescence intensity (542 nm) of AS‐nFAp:Gd/Tb NPs under different synthetic times; (E), The fluorescence intensity of AS‐nFAp:Gd/Tb on the 542 nm with different concentration molar ratio of Gd^3+^ ions to Ca^2+^ ions and Tb^3+^ ions to Ca^2+^ ions; F, The fluorescence intensity of AS‐nFAp:Gd/Tb on the 542 nm with different concentration molar ratio of AS1411 to Ca^2+^

Both the FTIR spectra (Figure 2A) of AS‐nFAp:Gd/Tb and AS1411‐free nFAp:Gd/Tb showed the broad bands at approximately 1641 and 3430 cm^‐1^, which were attributed to the absorbed water in the HAp.^37^ The bands at about 1420‐1450 cm^‐1^ and 871 cm^‐1^ were ascribed to the carbonate groups included in the HAp structure. In addition, the characteristic peaks of PO_4_^3‐^ (centred at around 960, 474, 1110, 1037, 605 and 563 cm^‐1^) in HAp appeared in both AS‐nFAp:Gd/Tb and AS1411‐free nFAp:Gd/Tb.^36^, ^38^ However, the C = O band at about 1705 cm^‐1^ was only found in the AS‐nFAp:Gd/Tb, while it was absent in the nFAp:Gd/Tb, which was possibly obtained from DNA aptamer AS1411.^39^ The FTIR spectra of AS‐nFAp:Gd/Tb (Figure S1) consisting of different concentrations of AS1411 also confirmed the presence of C = O band in AS‐nFAp:Gd/Tb. Furthermore, based on the result of capillary electrophoresis (Figure 2B), a specific peak found on the AS1411 aptamer was not detected on the supernatant collected after centrifugation of the synthesized samples with Ca^2+^ ions bound to AS1411, indicating that the AS1411 was successfully coupled to the prepared nanoparticles.

With the increasing concentration of doped AS1411, the zeta potential values of AS‐nFAp:Gd/Tb were gradually decreased to negative values (Figure 2C). The particle size of AS1411‐free nFAp was optimized by the doping of AS1411, and the particle size of AS‐nFAp:Gd/Tb was smaller and tended to be stable when the molar ratio of AS1411 to Ca^2+^ was 1/1000, indicating that AS1411 played a crucial role in mediating the growth and the nucleation of nHAp. In addition, the particle sizes and fluorescence intensity of the prepared AS‐nFAp:Gd/Tb at different reaction times were displayed in Figure 2D. Under the reaction time of 6 hours, the particle size of the NPs was the smallest (68.06 nm), followed by the size of the NPs (105.71 nm) under the reaction time of 12 hours, which had the highest fluorescence emission intensity at the wavelength of 542 nm. Considering the concentration quenching effect of the interaction among doped ions,^40^ the influence of the doping concentrations of Gd^3+^ ions and Tb^3+^ ions in the synthetic system on the fluorescence intensity of the NPs was also explored (Figure 2E). When the molar ratio of Tb^3+^ ions to Ca^2+^ ions was 15% (Tb^3+^/Ca^2+^ = 15%), the NPs had the highest fluorescence intensity with the constant concentration of Gd^3+^ ions. Moreover, the fluorescence intensity of the prepared AS‐nFAp:Gd/Tb NPs was the highest in the system with the Gd^3+^ ions to Ca^2+^ ions molar ratio of 5% (Gd^3+^/Ca^2+^ = 5%).

### 3.2 Fluorescence properties of AS‐nFAp:Gd/Tb

**FIGURE 3:**
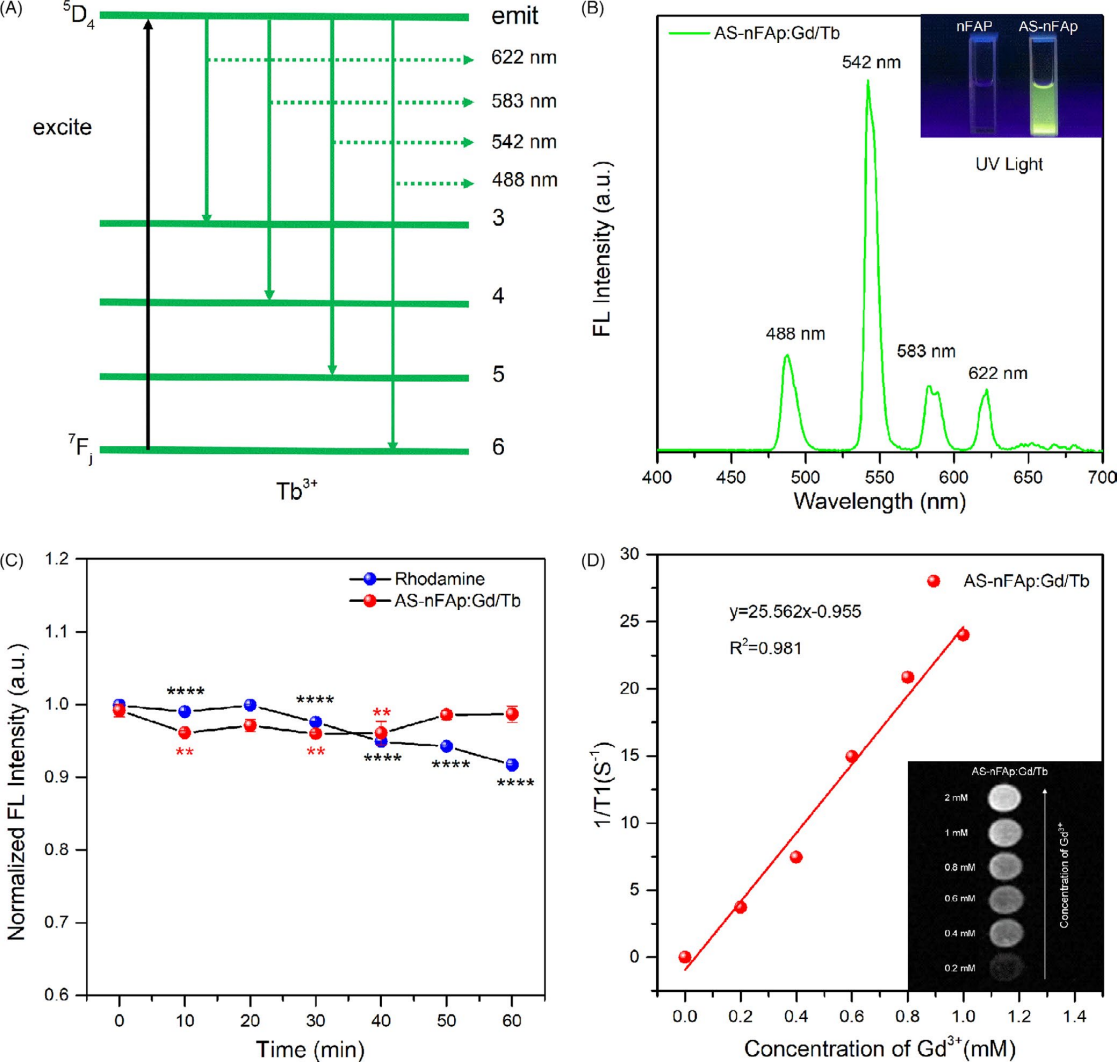
The fluorescence and MRI properties of the prepared AS‐nFAp:Gd/Tb NPs: (A), Schematic energy level diagram of Tb^3+^ ions; (B), The PL emission spectrum of AS‐nFAp:Gd/Tb NPs excited by 285 nm. Inset: the images of AS‐nFAp:Gd/Tb and AS1411‐free nFAp:Gd/Tb NPs excited by UV‐light at room temperature; (C), The photostability of AS‐nFAp:Gd/Tb NPs, *P < .05, **P < .01, ****P < .0001, *compare with the base time points. Data are shown as mean ± SD (n = 3); (D), The curve of relaxation values and T1‐weighted MRI images (inset) of AS‐nFAp:Gd/Tb at various concentrations in buffer solution.

The fluorescent nHAps doped with lanthanide (Ln^3+^) ions were reported to have a high‐performance optical application.^20,41^ Due to similar ionic radius and coordination environment of Tb^3+^ ions with Ca^2+^, Tb^3+^ could be doped into nHAp by replacing Ca^2+^ or inserting Ca^2+^ vacancies.^41^, ^42^ The PL emission spectrum (Figure 3B) of AS‐nFAp:Gd/Tb displayed narrow emission bands of four emission peaks (488 nm, 542 nm, 583 nm and 622 nm), corresponding to the 5D_4_→7F_j_ (j = 6, 5, 4, 3) transition of Tb^3+^ (Figure 3A), under an excitation wavelength of 285 nm. The fluorescence intensity of AS‐nFAp:Gd/Tb was increased with the increase of AS1411 doping amount (Figure 2F). The result of ICP‐MS indicated that the doping efficiencies of the doped Ln^3+^ ions in AS‐nFAp:Gd/Tb and AS1411‐free nFAP:Gd/Tb were similar (Table S1). However, AS‐nFAp:Gd/Tb emitted a strong green fluorescence under UV‐light, while AS1411‐free nFAP:Gd/Tb showed no visible green fluorescence (inset in Figure 3B). The reason may be that the surface defects of the nHAp could be effectively reduced by the adsorption of AS1411 on the surface for Ca^2+^ ions, thereby increasing the quantum field^43^, while for AS1411‐free nFAp:Gd/Tb NPs, there is no ligand on the surface to bind with Ca^2+^ ions to reduce the surface defects.

To explore the photostability of AS‐nFAp:Gd/Tb, the fluorescence intensity of aqueous solution of AS‐nFAp:Gd/Tb and rhodamine at different times within 1 hour was tested by a fluorescence spectrophotometer, and the measurement was repeated for 3 times at each time point. The fluorescence intensity of the rhodamine solution was gradually decreased with the prolonged time of the laser light (Figure 3C), and the difference was statistically significant, while the fluorescence intensity of the AS‐nFAp:Gd/Tb solution remained relatively stable, proving the better photostability of the fluorescent nano‐probe compared with ordinary fluorescent dyes.

### 3.3 In vitro MRI imaging

The Gd^3+^‐based systems have been widely designed and characterized as T1‐weighted MRI contrast agents in MRI imaging^44^, ^45^, ^46^. Thus, the potential of AS‐nFAp:Gd/Tb for MRI imaging was investigated. The prepared AS‐nFAp:Gd/Tb was dissolved in ultrapure water. Then, the T1‐weighted MRI images were evaluated on an MRI system, and the relaxation values were also measured. With the increasing dose of Gd^3+^, a significant dose‐dependent colour change and a good linear correlation change in relaxation values were observed (Figure 3D), proving the good MRI imaging potential of the prepared AS‐nFAp:Gd/Tb.

### 3.4 In vitro fluorescence imaging of cells

**FIGURE 4:**
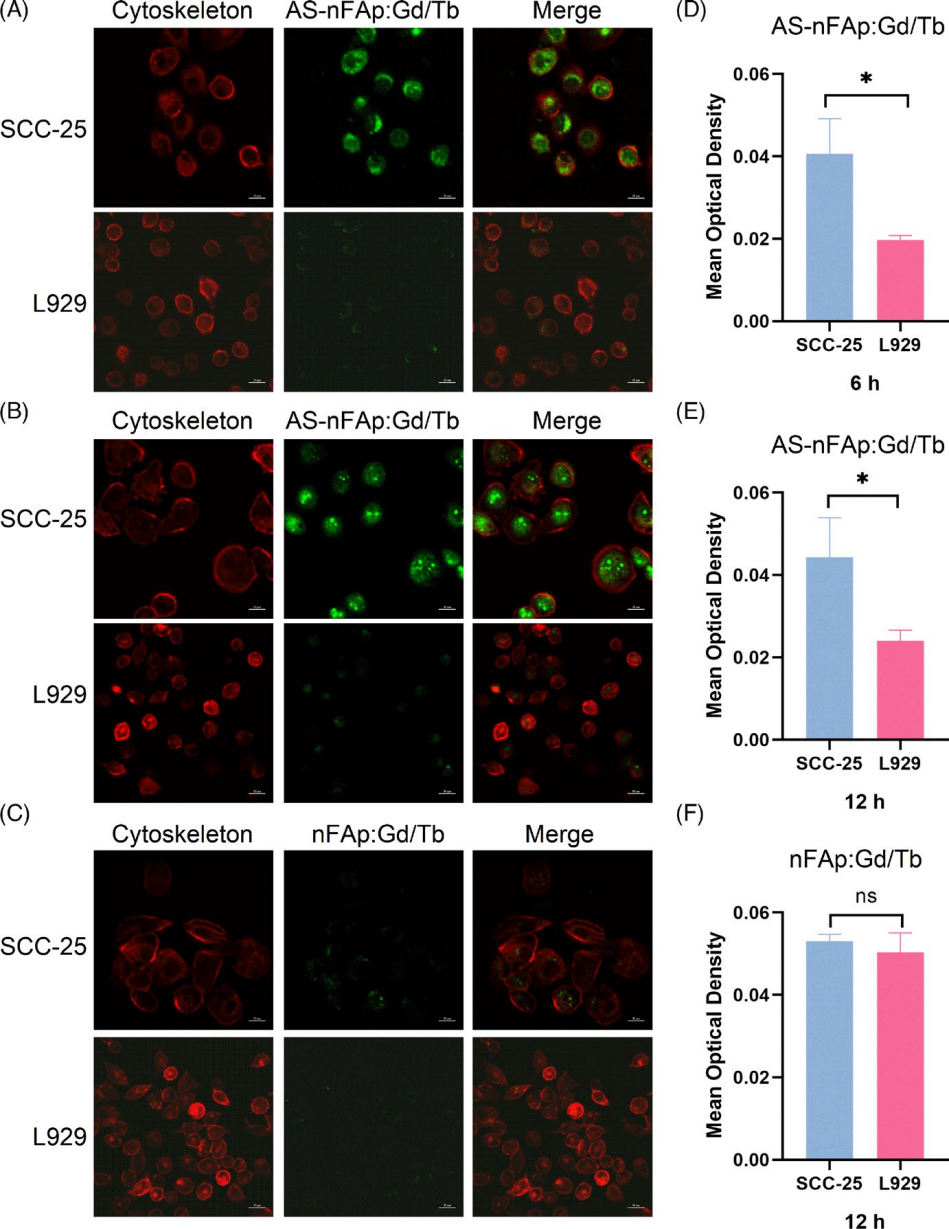
Cellular uptake of AS‐nFAp:Gd/Tb. (A), CLSM images of SCC‐25 and L929 cells after incubated with AS‐nFAp:Gd/Tb at 100 μg/mL for 6 h; (B), CLSM images of SCC‐25 and L929 cells after incubated with AS‐nFAp: Gd/Tb at 100 μg/mL for 12 h; (C), CLSM images of SCC‐25 and L929 cells after incubated with AS1411‐free nFAp:Gd/Tb at 100 μg/mL for 12 h; (D‐F), Statistical analyses of mean optical density in CLSM images. *P < .05, data are shown as mean ± SD (n = 3)

The potential of AS‐nFAp:Gd/Tb in fluorescence imaging applications was tested by SCC‐25 cells and L929 cells with differential expression of nucleolin. The expression of nucleolin in SCC‐25 cell line was confirmed to be significantly higher than that in L929 cell line by CLSM (Figure S2). Firstly, the cytotoxicity of AS‐nFAp:Gd/Tb was explored by a CCK‐8 kit compared with AS1411‐free nFAp:Gd/Tb. The two NPs had no obvious cytotoxicity to the cells in connection with the good biocompatibility of nHAp (Figure S3), indicating that the cytotoxicity of the NPs was not affected by the doped Ln^3+^ ions. Then, the AS‐nFAP:Gd/Tb of the same concentration was used to be co‐cultured with SCC‐25 cells and L929 cells, respectively, to determine the trait of the NPs’ targeted imaging. The successful entrance of AS‐nFAp:Gd/Tb into the nucleus of SCC‐25 (Figure 4A,B) was confirmed after incubated for 6 and 12 hours, respectively. However, the weak fluorescence was observed in L929 cells, and the mean optical density of SCC‐25 and L929 cells was statistically different at two time periods (Figure 4D‐E), proving the ability of tumour‐targeted imaging of the AS‐nFAp:Gd/Tb. Furthermore, the same concentration of AS1411‐free nFAp:Gd/Tb was used to be co‐cultured with the above two cell lines for 12 hours. No obvious green fluorescence was observed in both SCC‐25 cells and L929 cells under the same excitation light (Figure 4C, F), proving that the presence of the AS1411 could endow the NPs the ability to target tumour cells by elevated imaging performance.

### 3.5 Preparation of DOX@AS‐nFAp:Gd/Tb

**FIGURE 5:**
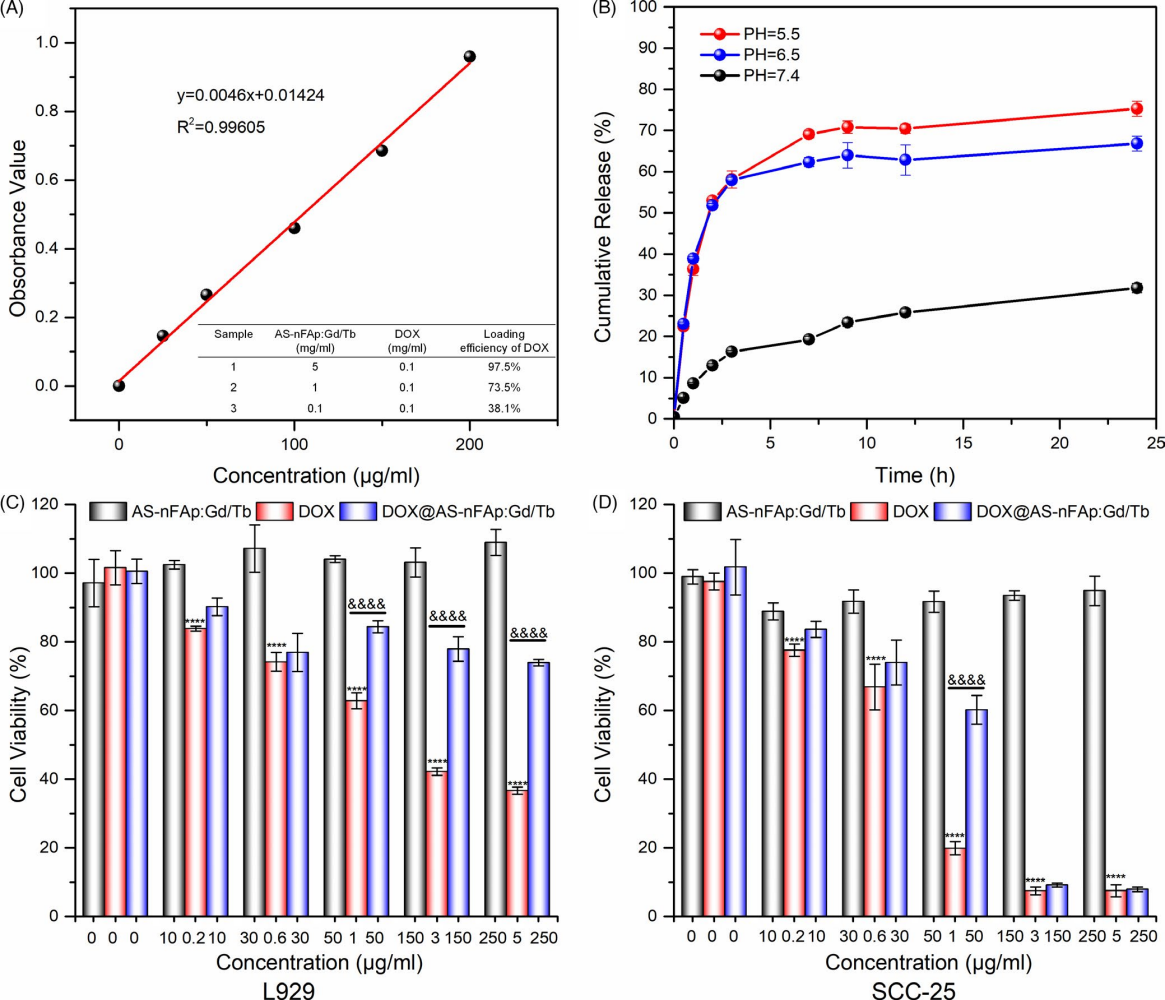
Drug loading capacity and cytotoxicity in vitro. (A), The standard dilution curve of DOX at different concentrations, table inserted shows the loading efficiency of DOX in DOX@AS‐nFAp:Gd/Tb; (B), The release profiles of DOX from DOX@AS‐nFAp:Gd/Tb in different pH buffers (n = 3); (C) and (D), The cell viability of L929 and SCC‐25 cells after treated with AS‐nFAp:Gd/Tb, DOX and DOX@AS‐nFAp:Gd/Tb at different concentrations for 24 h (the content of DOX in DOX@AS‐nFAp:Gd/Tb group was equivalent to the pure DOX group). ****P < .0001, ^&&&&^ *P* < .0001, * represents different concentrations vs ctrl in DOX group, & represents DOX group vs DOX@AS:nFAp:Gd/Tb at the same concentration, data are shown as mean ± SD (n ≥ 3)

The chemotherapy drug DOX was loaded to AS‐nFAp:Gd/Tb to explore the application of the NPs as a drug delivery system. Firstly, the increasing zeta potential of DOX@AS‐nFAp:Gd/Tb compared with AS‐nFAp:Gd/Tb (Figure S4) proved the successful loading of DOX in DOX@AS‐nFAp:Gd/Tb. Secondly, the loading efficiency and loading content of DOX@AS‐nFAp:Gd/Tb were investigated by a UV‐vis absorption spectroscopy at the wavelength of 485 nm. DOX was incorporated with AS‐nFAp:Gd/Tb at different molar ratios to quest the excellent loading condition. As shown in the standard dilution curve of DOX and the inserted table (Figure 5A), the loading efficiency and loading content of DOX@AS‐nFAp:Gd/Tb were up to 97.5% and 19.5 mg/g when the mass concentration ratio of DOX to AS‐nFAp:Gd/Tb was 1:50. Due to the electrostatic adsorption of DOX and AS‐nFAp:Gd/Tb, DOX can be loaded on AS‐nFAp:Gd/Tb with such a high efficiency. In addition, the release kinetics of DOX in DOX@AS‐nFAp:Gd/Tb were explored by incubating in PBS of different pH environments (pH = 5.5, 6.5 and 7.4) and monitored for 24 hours. The significantly accelerated release of DOX at pH5.5 and pH6.5 was observed compared with pH7.4 (Figure 5B), indicating that DOX@AS‐nFAp:Gd/Tb could efficiently release DOX under acidic environment, which may be due to the rapid degradation of nHAp in an acidic environment and the decrease in the adsorption capacity of nHAp to DOX.^47^ The superior drug loading capacity and pH‐induced drug release of DOX@AS‐nFAp:Gd/Tb demonstrated the potential of an attractive nanocarrier for drug delivery.

### 3.6 Cell viability and cell apoptosis analysis

The potential of DOX@AS‐nFAp:Gd/Tb for targeted tumour therapy was further investigated by a CCK‐8 kit in SCC‐25 cells and L929 cells. The pure DOX group exhibited dramatically dose‐dependent cytotoxicity to SCC‐25 cells and L929 cells, and DOX@AS‐nFAp:Gd/Tb showed similar cytotoxicity to SCC‐25 cells with DOX group at the high dose of DOX, while DOX@AS‐nFAp:Gd/Tb displayed lower toxicity to L929 cells than pure DOX group (Figure 5C,D). These results further demonstrated that DOX@AS‐nFAp:Gd/Tb had the potential to selectively enter tumour cells and then release DOX, thereby protecting the normal cells simultaneously. Based on the immunofluorescence analyses (Figure S5) on apoptosis‐related proteins, as compared with pure DOX group, the increased level of Bcl‐2 protein and the decreased level of Bax and Caspase‐3 proteins in L929 cells treated with DOX@AS‐nFAp:Gd/Tb indicated that DOX@AS‐nFAp:Gd/Tb is less toxic to normal cells. As for SCC‐25 cells, the cell apoptosis analysis also demonstrated the obvious toxicity of DOX@AS‐nFAp:Gd/Tb to tumour cells and good biocompatibility of AS‐nFAp:Gd/Tb NPs. Overall, the prepared DOX@AS‐nFAp:Gd/Tb exhibited a selective feature and a potential clinical application in tumour treatment.

### 3.7 In vivo anti‐tumour study

**FIGURE 6:**
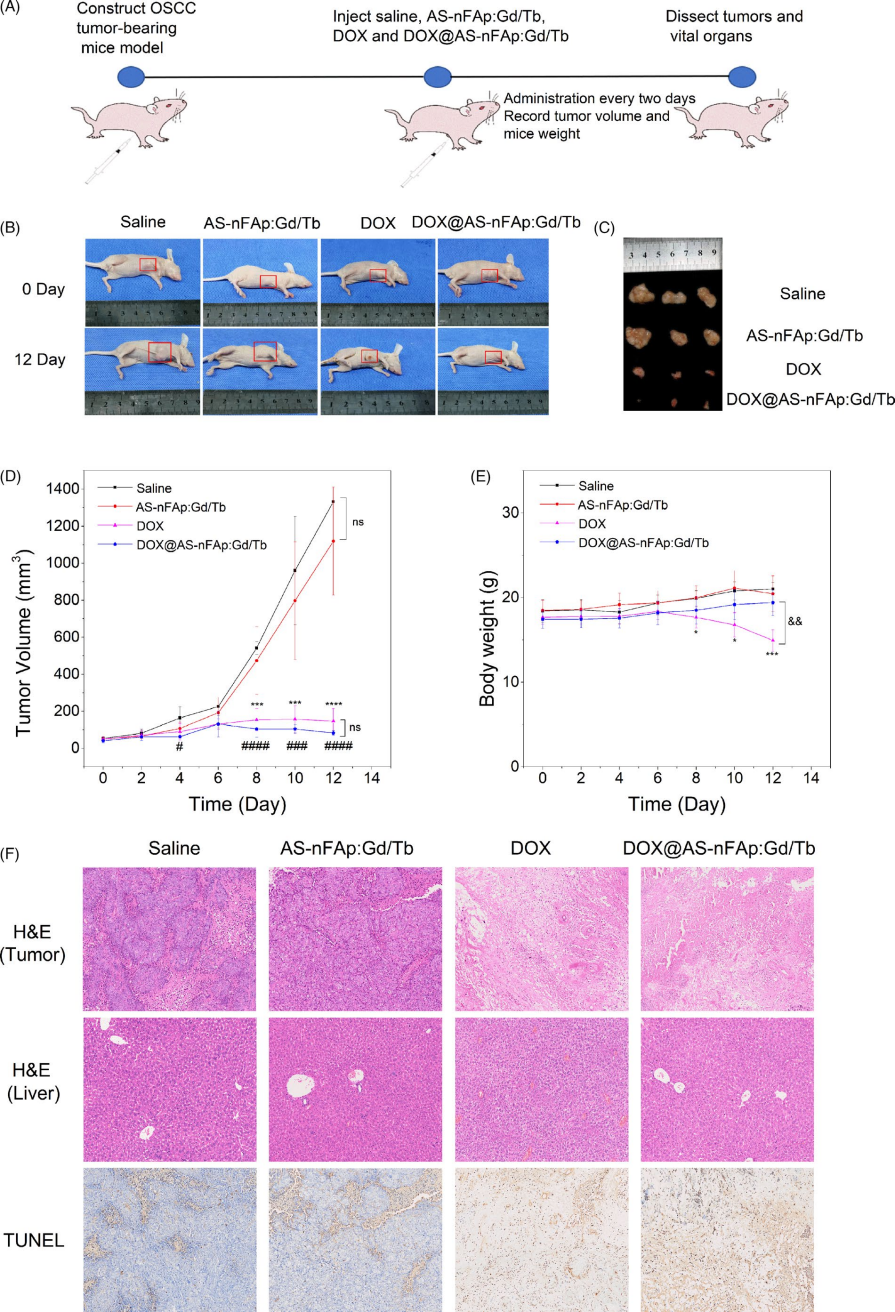
The anti‐tumour effect of DOX@AS‐nFAp:Gd/Tb in vivo study. (A), The oral squamous cell carcinoma (OSCC) tumour‐bearing mice model (mice injected with SCC‐25 cells) was constructed to investigate the application of DOX@AS‐nFAp:Gd/Tb in vivo experiments; (B), The photographs of tumour‐bearing mice in different treatment groups (saline, AS‐nFAp:Gd/Tb, DOX and DOX@AS‐nFAp:Gd/Tb) at the first day and the 12th day; (C), Ex vivo tumour images at the 12th day. (D), The curves of tumour volumes during different treatments; (E), The curves of body weights of tumour‐bearing mice during different treatments; (F), The H&E staining of tumour issues and liver, and the TUNEL staining of tumour issues in 4 different groups after 12‐day treatments (The magnification is 20 × 4). *P < .05, ***P < .001, ****P < .0001, ^###^ *P* < .001, ^####^ *P* < .0001, ^&&^ *P* < .01, * represents DOX group vs saline group at the same day, ^#^ represents DOX@AS‐nFAp: Gd/Tb group vs saline group at the same day, ^&^ represents DOX group vs DOX@AS‐nFAp:Gd/Tb group at the same day. Data are shown as mean ± SD (n ≥ 3)

As illustrated from Figure 6A, in order to systematically analyse the therapeutic effect of DOX@AS‐nFAp:Gd/Tb, 4 groups of randomly assigned SCC‐25 tumour‐bearing nude mice were injected into the paracancerous tissues with saline, AS‐nFAp:Gd/Tb, DOX and DOX@AS‐nFAp:Gd/Tb, respectively, when the tumours grew to 50 mm^3^. Figure 6B,C showed that the groups of DOX and DOX@AS‐nFAp:Gd/Tb significantly inhibited the growth of the tumours. The curves of Figure 6D also demonstrated that both the DOX and DOX@AS‐nFAp:Gd/Tb groups exhibited a great inhibition on the average tumour volume (from the 8th of treatment). In contrast, the tumours in groups of saline and AS‐nFAp:Gd/Tb continued to grow during the period of treatment. Although the free DOX also showed a significant inhibition on the tumour volume, the distinct body weight loss of mice was observed during treatment in the free DOX group due to the severe systemic toxicity of DOX,^48^ while the body weight in DOX@AS‐nFAp:Gd/Tb group remained stable (Figure 6E). This indicated excellent biocompatibility of DOX@AS‐nFAp:Gd/Tb, which could reduce side effects of DOX.

In addition, the anti‐tumour effect of DOX@AS‐nFAp:Gd/Tb was further studied by the H&E and TUNEL staining of tumour tissues in 4 groups after 12‐day treatments (Figure 6F). No obvious changes in cell morphology and tissue morphology were observed in the saline group and AS‐nFAp:Gd/Tb group, implying the good biosafety of AS‐nFAp:Gd/Tb. However, in the groups of DOX and DOX@AS‐nFAp:Gd/Tb, a large number of necrotic tumour tissues were observed due to the toxicity of DOX. The apoptotic cells were usually detected by positive TUNEL staining under light microscopy.^49^ From the TUNEL staining results, the tumours treated with pure DOX and DOX@AS‐nFAp:Gd/Tb had extensive regions of apoptotic cells (brown), proving that DOX@AS‐nFAp:Gd/Tb could effectively inhibit the proliferation of cancer cells and promote the cancer cells to apoptosis. The H&E staining of organs from tumour‐bearing mice in four different groups further confirmed the biosafety of DOX@AS‐nFAp:Gd/Tb. No obvious tissue damage and inflammation were observed in saline, AS‐nFAp:Gd/Tb and DOX@AS‐nFAp:Gd/Tb groups (Figure S6); conversely, the hepatocyte cytoplasmic porosity of the liver was noticed in the free DOX‐treated group (Figure 6F), which may be the toxic liver injury caused by chemotherapy.^50^ These results indicated that DOX@AS‐nFAp:Gd/Tb could effectively delivery DOX to tumour tissues and exert a highly effective tumour inhibition with the negligibly systemic toxicity simultaneously.

## 4 CONCLUSIONS

In this study, a co‐doped nFAp was successfully synthesized with AS1411 as a template by a one‐pot procedure so as to achieve the AS1411‐targeted fluorescence/MRI dual‐model imaging. In the presence of AS1411, the prepared AS‐nFAp:Gd/Tb possessed good monodispersity and excellent fluorescence/MRI imaging properties. In addition, the chemotherapy drug DOX was loaded on AS‐nFAp:Gd/Tb to construct a multifunctional nano‐probe that integrated diagnosis and treatment. The results in vitro confirmed that DOX@AS‐nFAp:Gd/Tb had a superior capacity of drug loading and an effective pH‐induced drug release ability. In vivo anti‐tumour experiments demonstrated an excellent anti‐tumour effect of DOX@AS‐nFAp:Gd/Tb without any obvious side effects on mice during the treatment. Overall, the DOX@AS‐nFAp:Gd/Tb prepared by the biomimetic strategy showed the outstanding capabilities of recognition and treatment on tumours and thus had a potential clinical application in the future.

## CONFLICTS OF INTEREST

No conflict of interest was declared in this article.

## AUTHOR CONTRIBUTIONS

Wenqing Zhang, Yuting Yang, Shuanglin Peng and Dexuan Xiao performed the experiments. Wenqing Zhang, Ronghui Zhou and Tingting Kong conducted statistical analysis. Wenqing Zhang wrote the main text. Bofeng Zhu and Ronghui Zhou revised the manuscript. Bofeng Zhu, Ronghui Zhou and Xiaoxiao Cai designed the work and provided the conception. All authors gave their final approval and agreed to be accountable for all aspects of the work.

## Supporting information

Figure S1

Figure S2

Figure S3

Figure S4

Figure S5

Figure S6

Table S1

## Data Availability

The data, supporting the findings of this work, are available from the corresponding author upon reasonable request.
