# Supplementary figures and images for: Aptamer‐mediated synthesis of multifunctional nano‐hydroxyapatite for active tumour bioimaging and treatment
Source: Cell Prolif. 2021 Aug 12;54(9):e13105. doi: 10.1111/cpr.13105 (PMC8450118; doi:10.1111/cpr.13105)

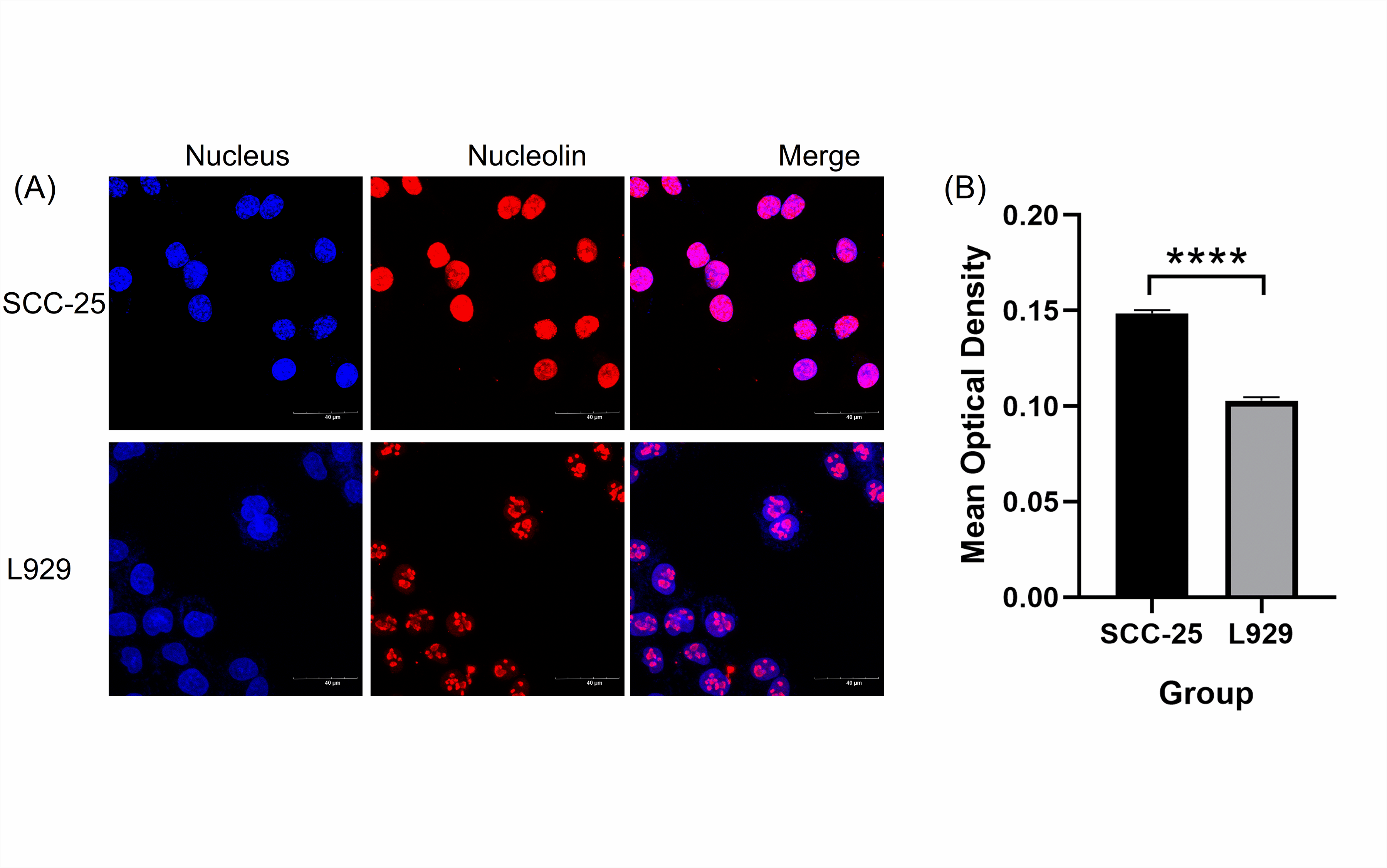

Supplement: Supplementary file 2 — Figure S2 [file CPR-54-e13105-s003.tif]

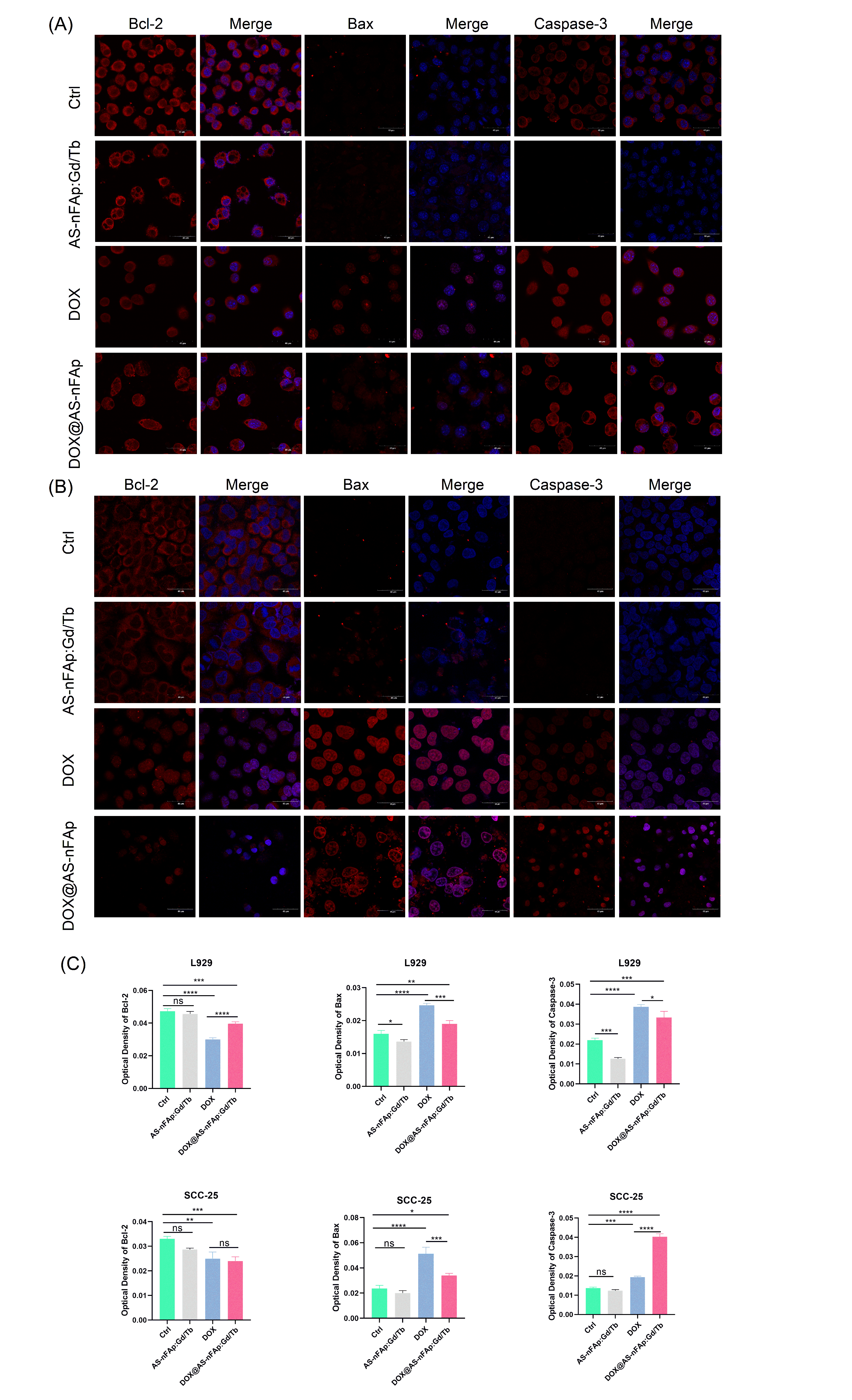

Supplement: Supplementary file 5 — Figure S5 [file CPR-54-e13105-s002.tif]

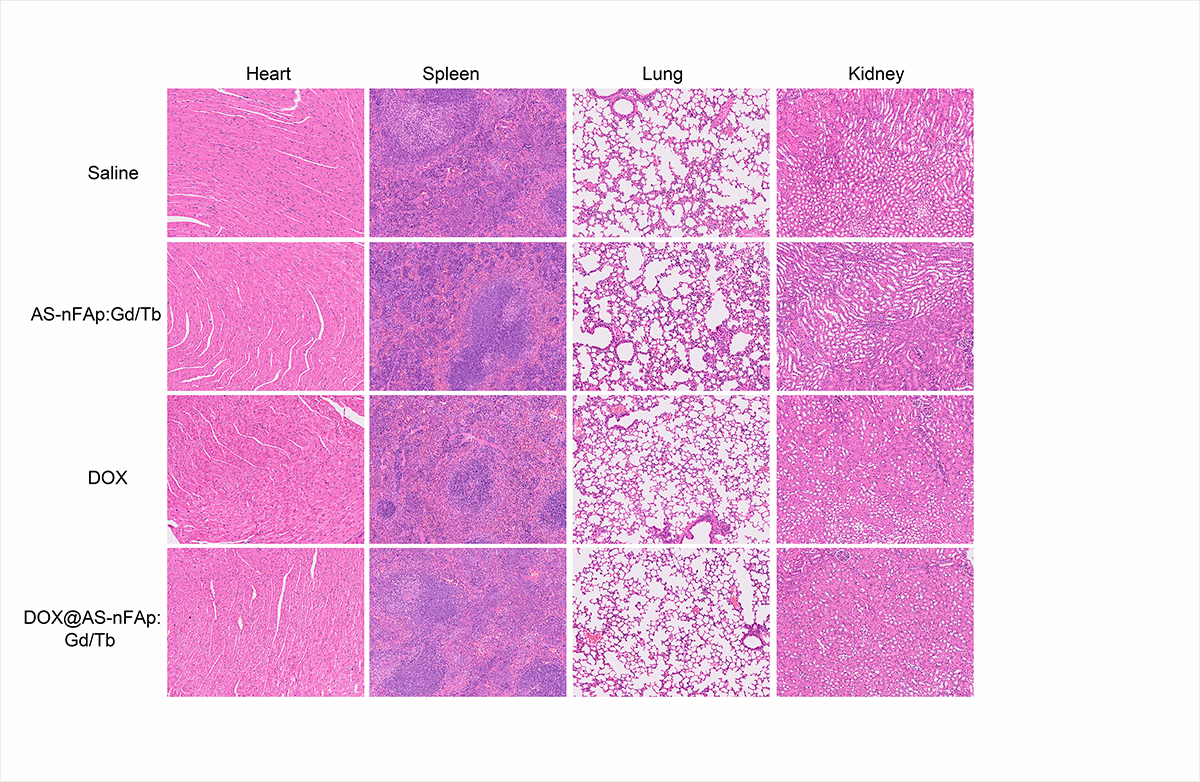

Supplement: Supplementary file 6 — Figure S6 [file CPR-54-e13105-s007.tif]
